# Intraoperative Fascia Tension as an Alternative to Component Separation. A Prospective Observational Study

**DOI:** 10.3389/fsurg.2020.616669

**Published:** 2021-02-23

**Authors:** Henning Niebuhr, Thomas Aufenberg, Halil Dag, Wolfgang Reinpold, Christian Peiper, Hans Martin Schardey, Marc Alexander Renter, Mohamed Aly, Dietmar Eucker, Ferdinand Köckerling, Jakob Eichelter

**Affiliations:** ^1^Hanse Hernia Centre, Hamburg, Germany; ^2^Clinic for Surgery, St. Elisabeth Hospital, Cologne, Germany; ^3^Clinic for Surgery, Groß Sand Hospital, Hamburg, Germany; ^4^Clinic for General, Visceral and Thoracic Surgery, Protestant Hospital, Hamm, Germany; ^5^Clinic for General, Visceral, Vascular and Endocrine Surgery, Agatharied Hospital, Hausham, Germany; ^6^Clinic for General and Visceral Surgery, St. Josef Hospital, Moers, Germany; ^7^Clinic for General, Visceral and Thoracic Surgery, Landshut-Achdorf Hospital, Landshut, Germany; ^8^Clinic for General, Visceral, Thoracic and Vascular Surgery, Canton Hospital Basel-Land, Liestal, Switzerland; ^9^Clinic for General, Visceral and Vascular Surgery, Vivantes Klinikum Spandau, Berlin, Germany; ^10^Department of Surgery, Division of General Surgery, Medical University of Vienna, Vienna, Austria

**Keywords:** abdominal wall hernia, incisional hernia, giant hernia, laparostoma, loss of domain, component separation, open abdomen, Fasciotens

## Abstract

Incisional hernias are common late complications of abdominal surgery, with a 1-year post-laparotomy incidence of about 20%. A giant hernia is often preceded by severe peritonitis of various causes. The Fasciotens® Abdomen device is used to stretch the fascia in a measurably controlled manner during surgery to achieve primary tension-free abdominal closure. This prospective observational study aims to clarify the extent to which this traction method can function as an alternative to component separation (CS) methods.

**Methods:** We included data of 21 patients treated with intraoperative fascia stretching in seven specialized hernia centers between November 2019 and August 2020.

**Results:** Intraoperatively-measured fascial distance averaged 17.3 cm (range 8.5–44 cm). After application of diagonal-anterior traction >10 kg for an average duration of 32.3 min (range 30–40 min), the fascial distance decreased by 9.8 cm (1–26 cm) to an average 7.5 cm (range 2–19 cm), which is a large effect (*r* = 0.62). The fascial length increase (average 9.8 cm) after applied traction was highly significant. All hernias were closed under moderate tension after the traction phase. In 19 patients, this closure was reinforced with mesh using a sublay technique.

**Conclusion:** This method allows primary closure of complex (LOD) hernias and is potentially less prone to complications than component separation (CS) methods.

## Introduction

Incisional hernia is a common late complication after abdominal surgery and is classified as an acquired abdominal wall defect. Incidence is about 20% 1 year after laparotomy (1). The risk for developing incisional hernia is multifactorial.

Consequences of incisional hernia include limitations in physical fitness, intestinal and organ functions, pain, and cosmetic impairment (2). These epidemiological data are of immense socio-economic importance, considering the high costs of follow-up.

**Figure 1:**
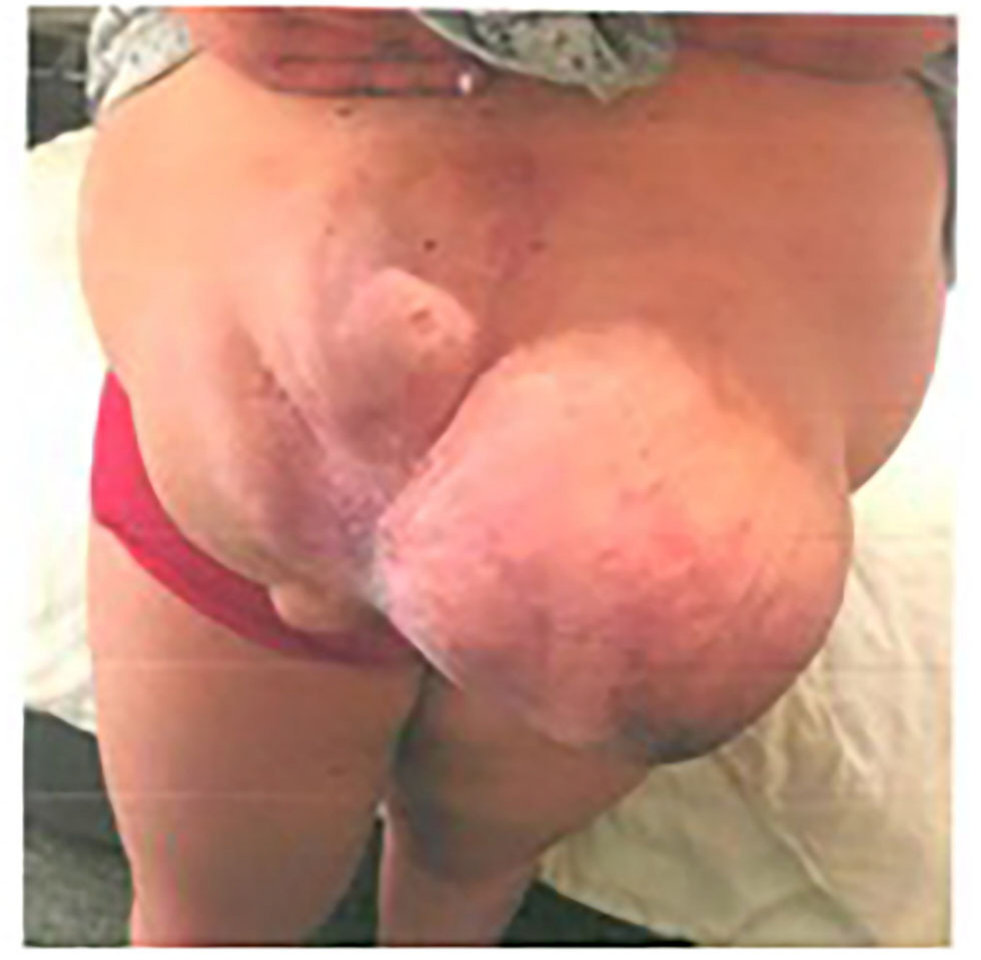
Schematic diagram of Fasciotens® by courtesy of Fasciotens GmbH 3.

Large incisional hernias have become an increasing challenge for general surgeons. The number of surgical procedures performed is increasing while patients become older and more obese. Hernias extending 10–25 cm from transverse and up to 30 cm from vertical incisions are not uncommon. Severe cases of giant hernias with loss of domain are referred to as complex hernias, but the definition of complex hernia is not consistent within the scientific literature (2, 3). Lateralization and shrinkage of the vertical abdominal muscles and the three oblique lateral muscles lead to hernia progression so that eventually, the hernia cannot be closed without tension. In this extreme form of hernia, surgical reconstruction of the abdominal wall is needed to avoid substantial complications, some of them life-threatening extreme form of hernia (see Figure 1).

A giant hernia is often preceded by severe peritonitis of various causes, often affecting patients suffering from multimorbidity and/or critical illness.

### Intraoperative Abdominal Wall Traction Procedures

Eucker et al. (4) described an innovative procedure to treat large abdominal wall hernias in 2017. An Abdominal Wall Expander System (AWEX) stretches the fascia anteriorly, enabling direct fascial closure. The case series reported on 10 patients, all of whom benefitted from the hernia repair. There were no recurrences over the median follow-up period of 21 months (range 7–36 months).

In 2019, Eickhoff et al. (5) assessed a porcine *in-vivo* model. They found that the abdominal wall can be stretched anteriorly in a similar way during temporary laparostomy, significantly reducing the force needed to reapproximate the fascial edges over an observation period of 48 h.

Based on these results, as well as good experience with the Fasciotens® Abdomen traction method used for the open abdomen, there seems to be an indication to apply this technique to complex hernia repair (6). The current study aims to clarify the extent to which the traction method can function as an alternative to component separation (CS) methods. CS is associated with severe complications, including a reported wound complication rate of up to 48.2% (7). Various other complications, such as ischemia, seroma, wound infection, etc., have been reported (7, 8).

We hypothesize that the new technique reported here may emerge as a useful alternative to conventional approaches to giant hernia repair, possibly avoiding the worst CS side effects.

## Materials and Methods

**Figure 2:**
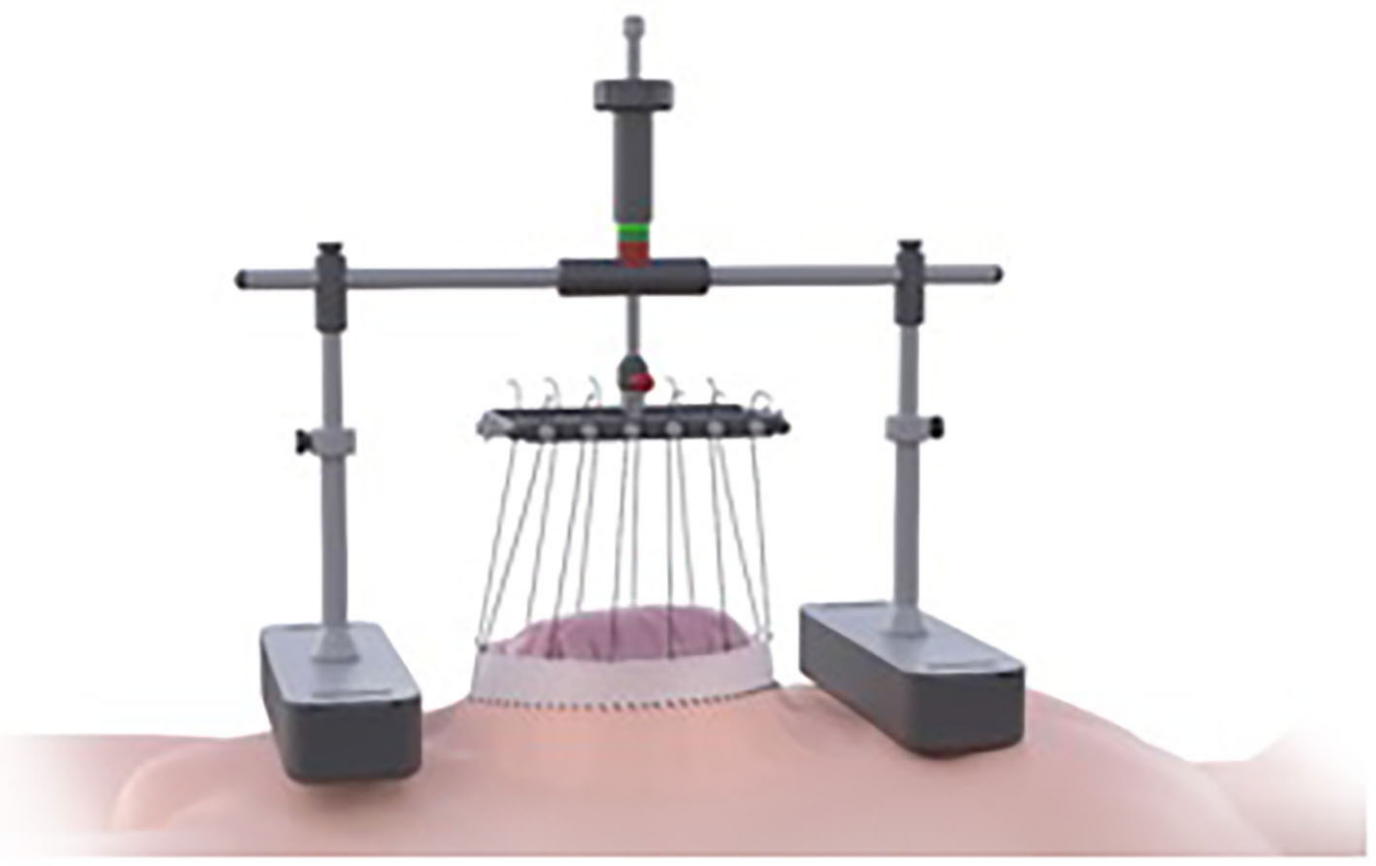
Patient 1 preoperative longitudinal hernia, private archive Dr. M. Renter 7.

This prospective multi-center observational study included data of 21 patients treated with the approved Fasciotens® Abdomen device, which stretches the fascia intraoperatively (see Figure 2). The study was conducted using standardized data forms from patients admitted to seven hospitals specializing in hernia repair between November 2019 and August 2020. All patients gave informed consent before the surgical procedure. Inclusion criteria were a measured fascial distance > 8 cm. Nineteen patients (90.5%) suffered from complex incisional hernia (loss of domain hernia). Seventeen patients (76.2%) had midline hernias, and four patients (19.0%) had transverse hernias. According to the classification of incisional hernias by the European Hernia Society (EHS), 19 hernias were W3 (>10 cm, 90.5%), and one hernia was W2 (<4–10 cm, 4.8%). One patient had a large primary hernia (W2) of the midline.

Patients receiving Botulinum toxin A (BTA) were pre-treated 4 weeks preoperatively and underwent ultrasound-guided injection of 200–300 I.U. BTA into the lateral abdominal muscles.

The Fasciotens® Abdomen device is used to stretch the fascia perpendicularly during surgery to achieve a primary tension-free abdominal closure and avoid the need for CS and potential complications. An external device applies anteriorly-directed traction on the abdominal wall structures (primarily the fascia), as shown in Figure 2.

For this study, the device was applied intraoperatively. The rectus sheath was opened and the space prepared for a sublay mesh augmentation. Epifascial dissection was limited to 2–3 cm per side to reduce invasiveness. Heavy suture (USP 1 or 2) was then placed using a U-suture technique along the fascia, with a bite depth of 1.5 cm and a bite width of 2–3 cm. In most cases, only the anterior rectus abdominis sheath was included in the sutures, and the hernial sac left intact. The alloplastic non-resorbable mesh was then placed in a typical sublay position. No surgical complications occurred directly caused by the traction, such as tear-out of the sutures.

**Figure 3:**
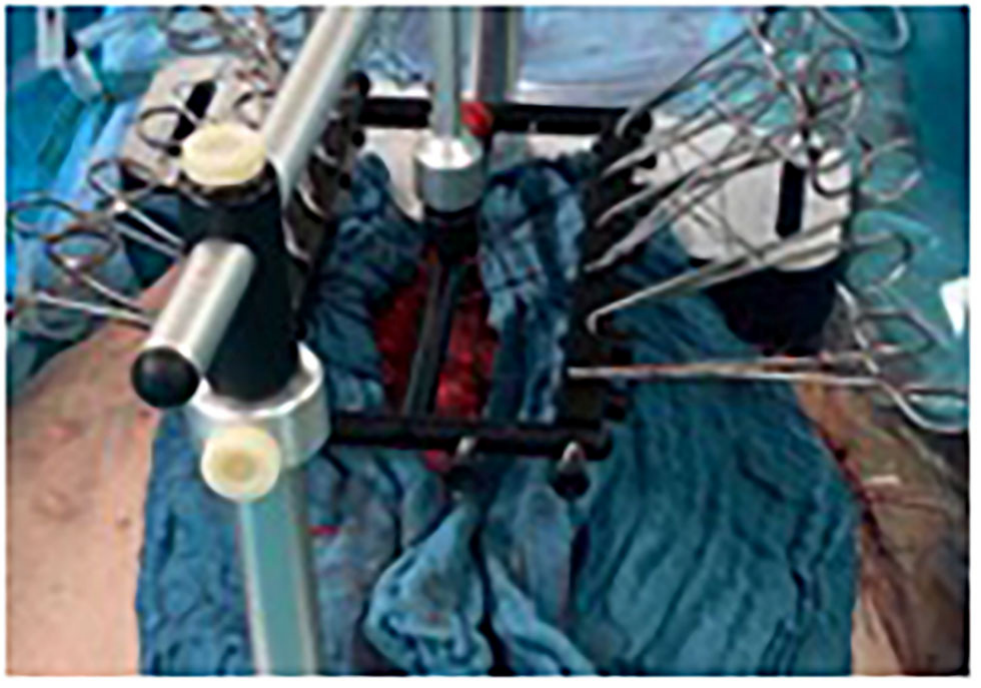
Patient 1 exemplary intraoperative Fasciotens application with clamp application for continuous retightening, private archive Dr. M. Renter 8.

The sutures were locked into the holding device, where they could be individually adjusted (see Figures 3, 8). A color indicator on top of the device estimates the cumulative tension applied. Thus, the abdominal wall is pulled anteriorly with continuous control over the traction force applied. A side result of the abdominal wall stretching is an increase in abdominal cavity volume. Tension is continuously adjusted by pulling and reattaching each suture or increasing the traction force with an adjustment handle on top of the device (above the color indicator). Traction was typically applied for 30 min, as longer times offered no further benefit.

Traction can be applied either anteriorly or diagonal-anteriorly, according to how sutures are attached to the device. In this study, only one patient received pure anterior traction, and the rest had diagonal-anterior traction.

In three cases, patients initially scheduled to receive traction did not. For these three, direct closure was deemed possible during surgery and performed without traction. These patients were not included in the study population.

Maximum fascial distance was always measured intraoperatively using a sterile tape ruler and under full patient relaxation. Measurements were taken before and after traction.

During the traction period, with total traction of 10 kg according to the color indicator, the sutures were re-tensioned and reattached to the device individually every 2 min.

The intra-abdominal pressure (IAP) was determined with continuous indirect intravesical measurement.

**Figure 4:**
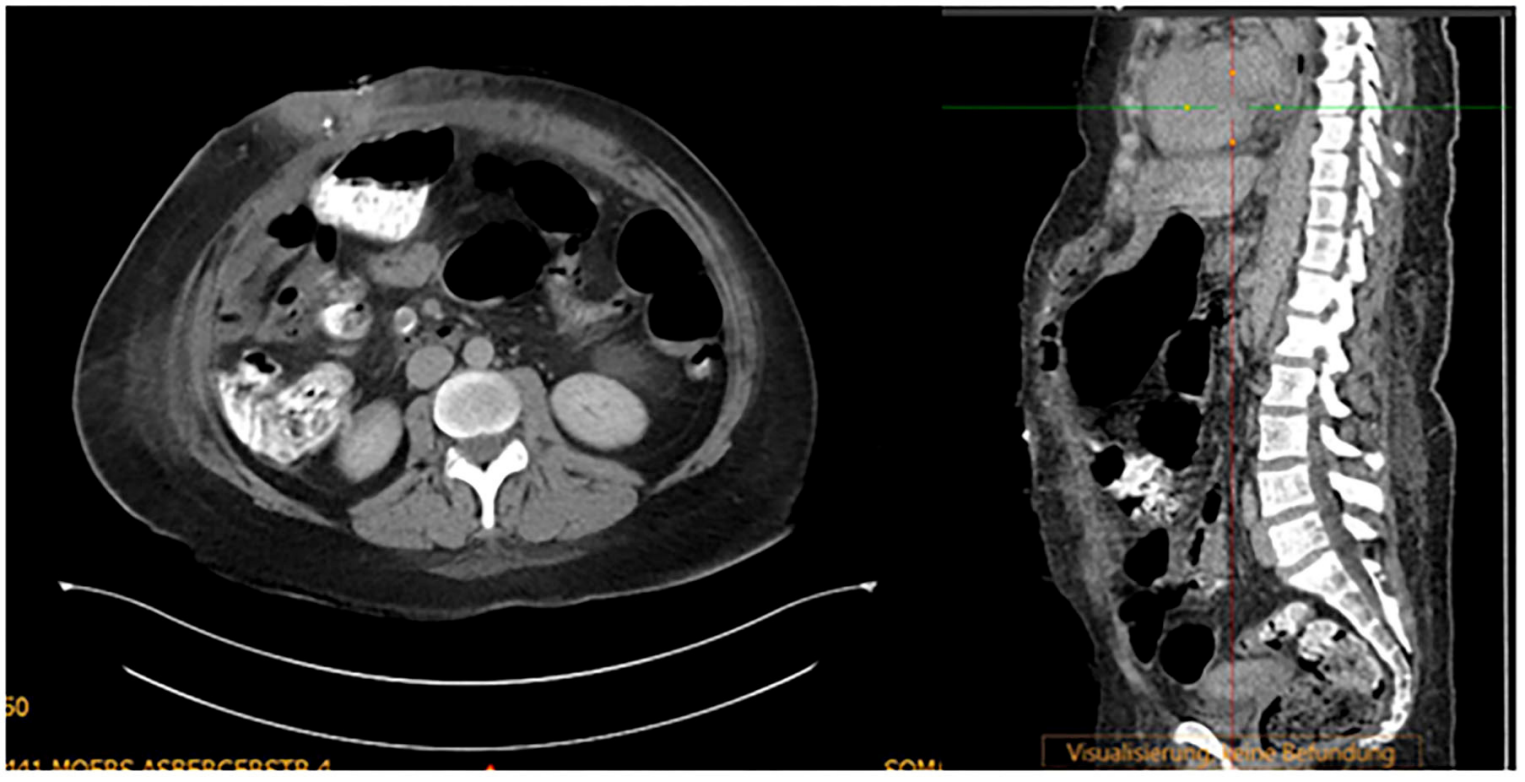
Patient 1 postoperative computer tomography, private archive Dr. M. Renter 8.

**Figure 5:**
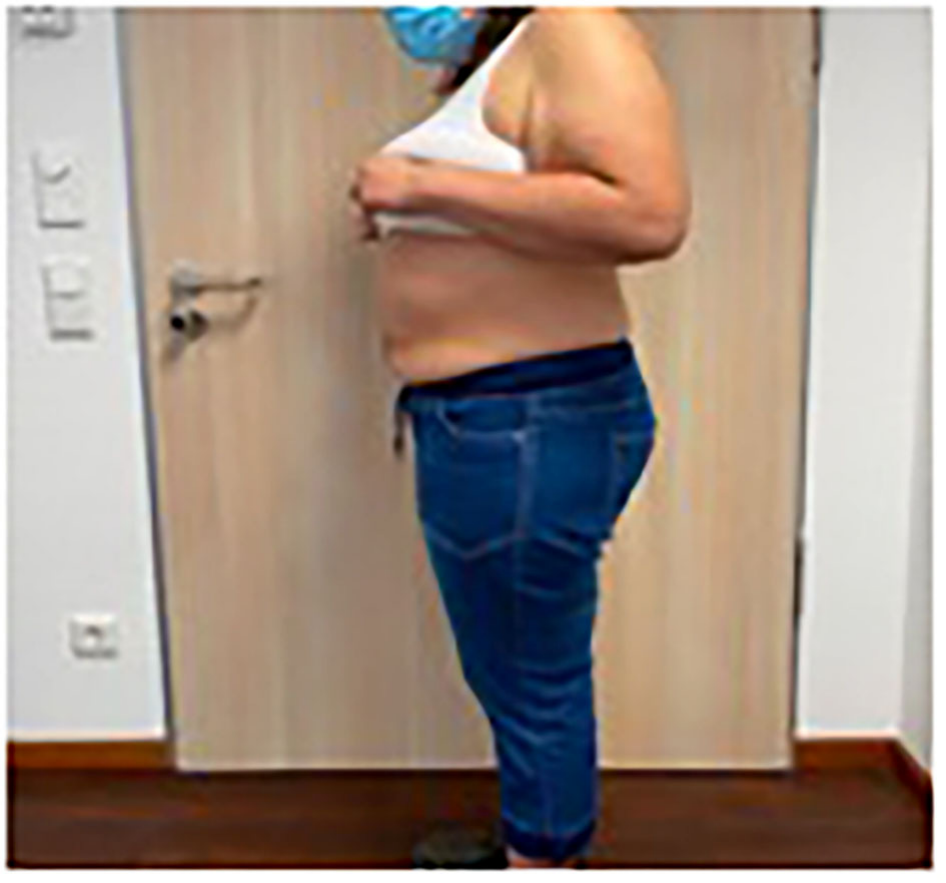
Patient 1 follow-up, private archive Dr. M. Renter 9.

Each patient returned for clinical and sonographic follow-up 2–4 weeks after discharge (see Figures 4, 5). Further follow-up (physical/telephone/video consultation) was planned at 3, 6, and 12 months.

A VAS scale (visual analog scale, 1–10) was used to assess pain immediately postoperatively and on the second postoperative day.

## Statistics

Statistical reports and analyses were carried out using the statistic software *IBM SPSS*^®^ *24 for Mac OSX*. The significance value was preemptively determined as α = 5%, so results with a *p*-value ≤ 0.5 were deemed significant.

Measurement parameters of baseline characteristics were specified as mean (*M*) with standard deviation (*SD*) and median (*Md*). Categorical variables were defined with frequency distribution (*n*) and percentage (%).

The Chi-squared test was used to analyze nominal scaled variables. The Wilcoxon signed-rank test was used to compare dependent samples, and the Mann–Whitney *U*-test was used to evaluate independent samples. Fisher's exact test was used to show statistical significance when comparing small sample sizes.

## Results

**Figure 6:**
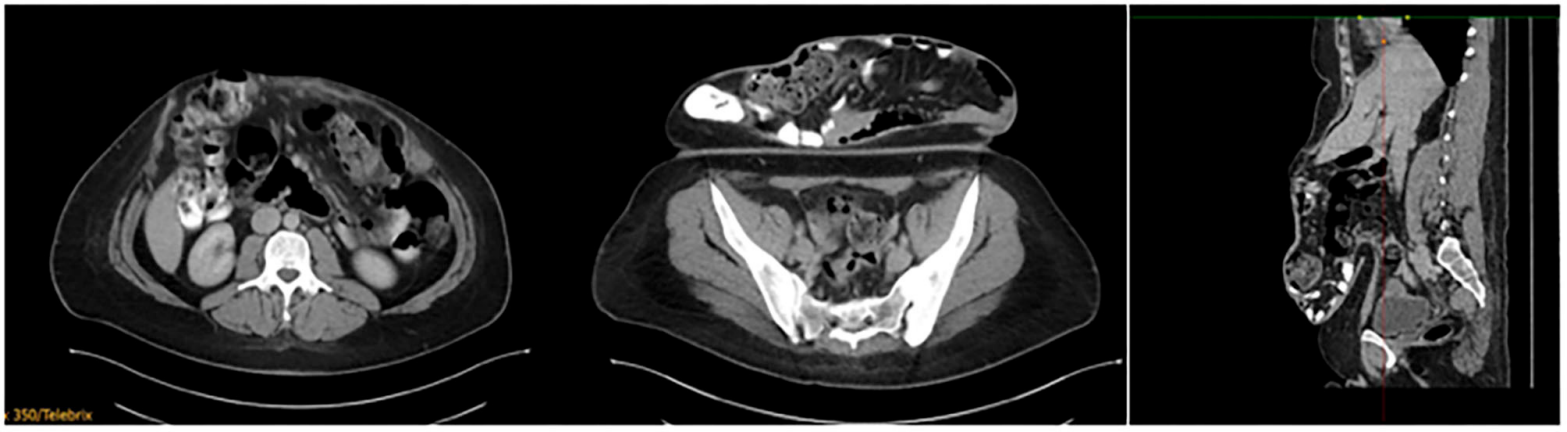
Patient 1 preoperative computer tomography, private archive Dr. M. Renter 7.

The average patient age was 58 years (range 33–77), with a gender ratio of 2.5 males: 1 female. ASA scores (assigned by the anesthesiologist) were III in 14 patients (66.7%) and II in 7 patients (33.3%) for this predominantly multimorbid collective. The body mass index (BMI) averaged 32.5 kg/m^2^ (range 20.3 −51.6). Fascial distance averaged 15.2 cm (range 7.7–44) on preoperative computer tomography (CT) scan or ultrasound (see Figure 6). Thirteen patients (61.9%) were treated with BTA 4 weeks before surgery.

**Figure 7:**
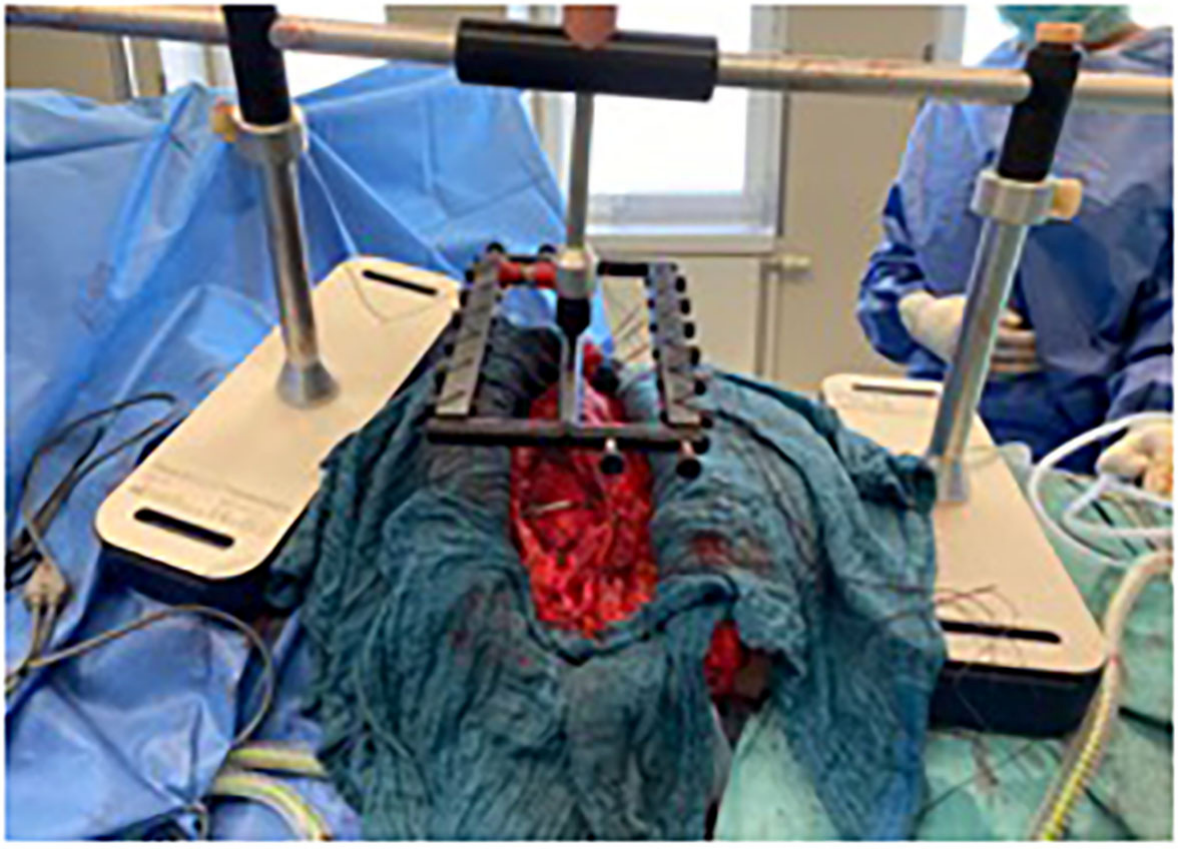
Patient 2 intraoperatively: Fasciotens transverse traction (transversely applied frame), private archive Prof. Dr. H. Niebuhr 9.

All patients were evaluated intraoperatively by the surgeons, and the subjective assessment for each included patient was that the hernia could not be closed by direct fascial approximation (See Figure 7). Thus, traction therapy was implemented. Component separation would have otherwise been necessary. One case underwent a Ramirez procedure as well. In this case, the traction method achieved approximation from 13 to 7 cm; however, the surgeon added a Ramirez maneuver to achieve a tension-free closure. This patient had a postoperative subcutaneous seroma treated with Vacuum-assisted closure (VAC).

Patients were always fully relaxed under anesthesia for this assessment, fascial distance measurements, and the traction phase.

Intraoperative measurements of fascial distance averaged 17.3 cm (range 8.5–44). After application of diagonal-anterior traction > 10 kg for an average of 32.3 min (range 30–40), the fascial distance decreased by 9.8 cm (range 1–26) to an average 7.5 cm (range 2–19), which represented a large effect (*r* = 0.62). In the 13 patients pre-treated with BTA, the average fascial distance decreased by 9.9 cm, from 16.1 to 6.2 cm. The preoperative fascial distance of all patients (average 15.2 cm, range 7.7–44 cm) measured on CT or ultrasound was smaller than the (relaxed) intraoperatively-measured distance (average 17.3 cm, range of 8.5–44 cm). Nevertheless, there was good reliability between preoperative imaging and intraoperative measurements (intraclass correlation coefficient, ICC = 0.94). The IAP was used for early detection of abdominal compartment syndrome. The IAP never exceeded 20 mmHg postoperatively and in all cases decreased to normal levels on the first postoperative day. Data on the IAP profiles were not collected.

There were highly significant gains of fascial length (on average 9.8 cm) with applied traction. All hernias were closed with moderate tension after the traction phase. In 19 patients, this closure was reinforced by a mesh inserted with a sublay technique.

Two closures were performed with an intraperitoneal onlay mesh (IPOM) for augmentation of the posterior wall.

One patient required VAC treatment due to a surgical site infection and reoperation on postoperative day 14 after a 5 cm fascial dehiscence. There were no other early complications. Three other patients required VAC therapy for subcutaneous wound healing disorders. One case needed a maximum of 11 VAC changes until secondary closure of skin and subcutaneous tissue occurred.

There were no other surgical complications. Two patients acquired nosocomial pneumonias postoperatively as well as cardiac decompensation with respiratory insufficiency and resulting Intermediate Care (IMC) treatment.

One patient suffered a postoperative pulmonary embolism, which was treated with anticoagulation.

None of the patients developed postoperative abdominal compartment syndrome.

Immediate postoperative pain was in the lower VAS range, with an average of 2.5 (range 0–6). The average hospital stay was 14.5 days (range 6–75) (see Table 1).

**Table 1:** Patient characteristics, measurements and statistical analysis (*N* = 21).

**1. Preoperative evaluation**	
Gender m/f	15/6 *N* = 21
Age [years] *M* ±*SD* (Range)	58.2 ± 12.2 (33–77) *N* = 21
*Md* (IQR)	59.0 (54–66)
BMI [kg/m^2^] *M* ±*SD* (Range)	32.5 ± 7.8 (20.3–51.6) *n* = 20
*Md* (IQR)	31.1 (26.5–36.9)
ASA	*N* = 21
I	0
II	7
III	14
IV	0
**2. Fascial measurements**	
Preoperative fascial distance on imaging [cm] *M* ±*SD* (Range)	15.2 ± 8.8 (7.7–44.0) *N* = 21
*Md* (IQR)	12 (10–17)
Intraoperative fascial distance before traction [cm] *M* ±*SD* (Range)	17.3 ± 8.6 (8.5–44.0) *N* = 21
*Md* (IQR)	15 (12–23)
Reliability of preoperative imaging compared to fascial distance measured intraoperatively (ICC)	0.94, 95% CI [0.82–0.98], *p* < 0.001^**^
Intraoperative fascial distance after traction [cm] *M* ±*SD* (Range)	7.5 ± 5.7 (2–19) *N* = 21
*Md* (IQR)	7 (3–9)
Net gain by traction [cm] *M* ±*SD* (Range)	9.8 ± 5.7 (1–26) *N* = 21
*Md* (IQR)	8 (7–12)
Net gain (Wilcoxon signed-rank test, one-tailed)	*p* < 0.001^**^ (effect *r* = 0.62)
Percentage of fascial gain through traction *M* ±*SD* (Range)	58.0 ± 22.2% (11–87%) *N* = 21
*Md* (IQR)	64.7% (43.8–75.0%)
**3. Surgical characteristics**	
Traction duration [min] *M* ±*SD* (Range)	32.5 ± 3.4 (30–40) *N* = 21
*Md* (IQR)	30 (30–35)
Intraoperative complications	none *N* = 21
Reconstruction method	*N* = 21
Sublay	18
IPOM	2
Ramirez + Sublay	1
Hospital stay [days] *M* ±*SD* (Range)	14.5 ± 15.2 (5–75) *N* = 21
*Md* (IQR)	9 (7–15)
Postoperative pain (VAS) *M* ±*SD* (Range)	2.5 ± 1.7 (0–6) *N* = 17
*Md* (IQR)	2 (1–3)
Postoperative Complications	8 (38.1%, 10 complications) *N* = 21
BTA vs. no BTA (Chi-Square-Test, χ, c.F.)	3 (23.1%) vs. 5 (62.5%), *p* = 0.164
Surgical complications (SSI *n =* 4, Fascial dehiscence <5 cm *n* = 1)	Four patients with surgical complications
Other postoperative complications(nosocomial pneumonia *n* = 2, respiratory insufficiency/cardiac decompensation *n* = 2, peripheral pulmonary artery embolism *n* = 1)	Five other complications
Botox yes/no	13/8 *N* = 21
BTA vs. no BTA (Mann–Whitney-*U*-Test, two-tailed)	*p* = 0.380
Preop. fascial distance on imaging (*Md* 10.0 vs. 13.5)	*p* = 0.258
Intraop. fascial distance before traction (*Md* 13.0 vs. 21.0)	*p* = 0.483
Intraop. fascial distance after traction (*Md* 4.0 vs. 8.5)	*p* = 0.763
Net gain by traction (*Md* 8.0 vs. 8.0)	*p* = 0.011^*^ (effect *r* = 0.60, *n* = 17)
Postoperative pain (*Md* 3 *n* = 9 vs. 1 *n* = 8)	*p* = 0.216
Hospital stay (*Md* 9 vs. 14.5)	
BTA vs. no BTA (Chi-Square-Test, χ, c.F.)Postoperative complications	3 (23.1%) vs. 5 (62.5%), *p* = 0.164

*M, mean; SD, standard deviation; Md, median; IQR, interquartile range; N, sample size; n, cases; r, effect size; p, significance value (probability); c.F., corrected by Fisher's exact test, ICC, intraclass correlation coefficient; preop., preoperative; intraop., intraoperative; vs., versus*.

** *p ≤ 0.01*,

* *p ≤ 0.05; α = 5%*.

*BMI, body mass index; ASA, ASA (American Society of Anesthesiologists) physical status classification system; IPOM, Intraperitoneal Onlay Mes; SSI, surgical site infection; BTA, botulinum toxin A*.

## Discussion

The reconstruction of complex hernias requires anatomical closure of the abdominal wall, which requires increased fascial length. Without sufficient gains in fascial length, the intra-abdominal pressure increases after abdominal closure. In our series, we gained a mean fascial length of 9.8 cm, which was highly significant. This increase is comparable to forms of CS reported by Majumder et al. who measured gains of 8.8 cm (ACS) and 10.2 cm (PCS) in a cadaver model (9). In our group, none of the patients developed abdominal compartment syndrome. Postoperative increases of IAPs up to 20 mmHg were tolerated and controlled the following day. In all cases, there was subsequent normalization of IAP on the first postoperative day. This is comparable to the results from Eucker et al. who also observed no cases of abdominal compartment syndrome after anterior abdominal wall stretching (4). Thus, two series have reported that ~30 min of intraoperative traction on the abdominal wall yields enough fascial stretching to enable direct closure of even sizeable abdominal wall defects. These closures are often supplemented with prosthetic mesh reinforcement in a sublay-technique, avoiding mesh to organ contact. In the current study, we considered fixation of the mesh to caudal anatomy (Cooper ligament or pubic symphysis) as suggested by McCulloch (10) to avoid herniation below the arcuate line.

To date, sufficient fascial length increases were only possible using various forms of CS (Ramirez, endoscopically assisted CS, TAR) (11–13). Because these procedures are invasive, however, complications are more frequent (seromas, infections, hematomas, abdominal wall necrosis, sensitivity disorders). In our collective, four patients experienced postoperative surgical complications worthy of intervention (19%). Surgical site infections (SSI) occurred in 4 patients and were treated with a VAC system. One of these patients had undergone a supplemental Ramirez procedure, and one had experienced a small fascial dehiscence (<5 cm) needing reoperation. Nielsen et al. reported a similar group of patients with a comparable complication rate, which underscores the high morbidity rate (14).

Nonetheless, it should be emphasized that fascial gains were sufficient to enable abdominal wall closure for very large hernias without complications (fascial dehiscence, reoperation) in 95.2% of cases (20 of 21).

In this multimorbid collective (two-thirds of the patients with preoperative ASA scores of 3), there were non-surgical postoperative complications in five patients (23.8%), including nosocomial pneumonia (*n* = 2), respiratory insufficiency of cardiac or respiratory origin (*n* = 2), and peripheral pulmonary embolism (*n* = 1).

The placement of the traction sutures requires limited fascial and subcutaneous dissection, so the subcutaneous wound area is significantly smaller than with the Ramirez technique, for example. There were no intra-abdominal complications in the current study. Compared to reports of subcutaneous complications using the Ramirez CS technique, our complication incidence appears low.

Furthermore, lateral abdominal wall integrity is not maintained using the various CS procedures. Simple stretching of the abdominal wall enables the lateral abdominal wall to remain intact. At the very least, there is no localized damage leading to fascial weakness; thus, lateral abdominal wall complications appear less likely over the long term. On the other hand, Daes et al. observed no reductions in abdominal wall thickness after endoscopic subcutaneous anterior CS, although no direct conclusions can be made regarding abdominal wall function (15). Concerning the short-term and long-term complications of both procedures, comparative follow-up examinations are necessary.

BTA has often been applied 4 weeks before surgery, for either traction methods or CS. We did not observe any advantages for patients pre-treated with BTA. This statement can only be made with reservations, however. This study has a divergent collective, multiple centers, and no exact study protocol regarding this treatment. A larger prospective randomized controlled trial would be necessary to evaluate BTA effects on traction effectiveness and/or CS.

**Figure 8:**
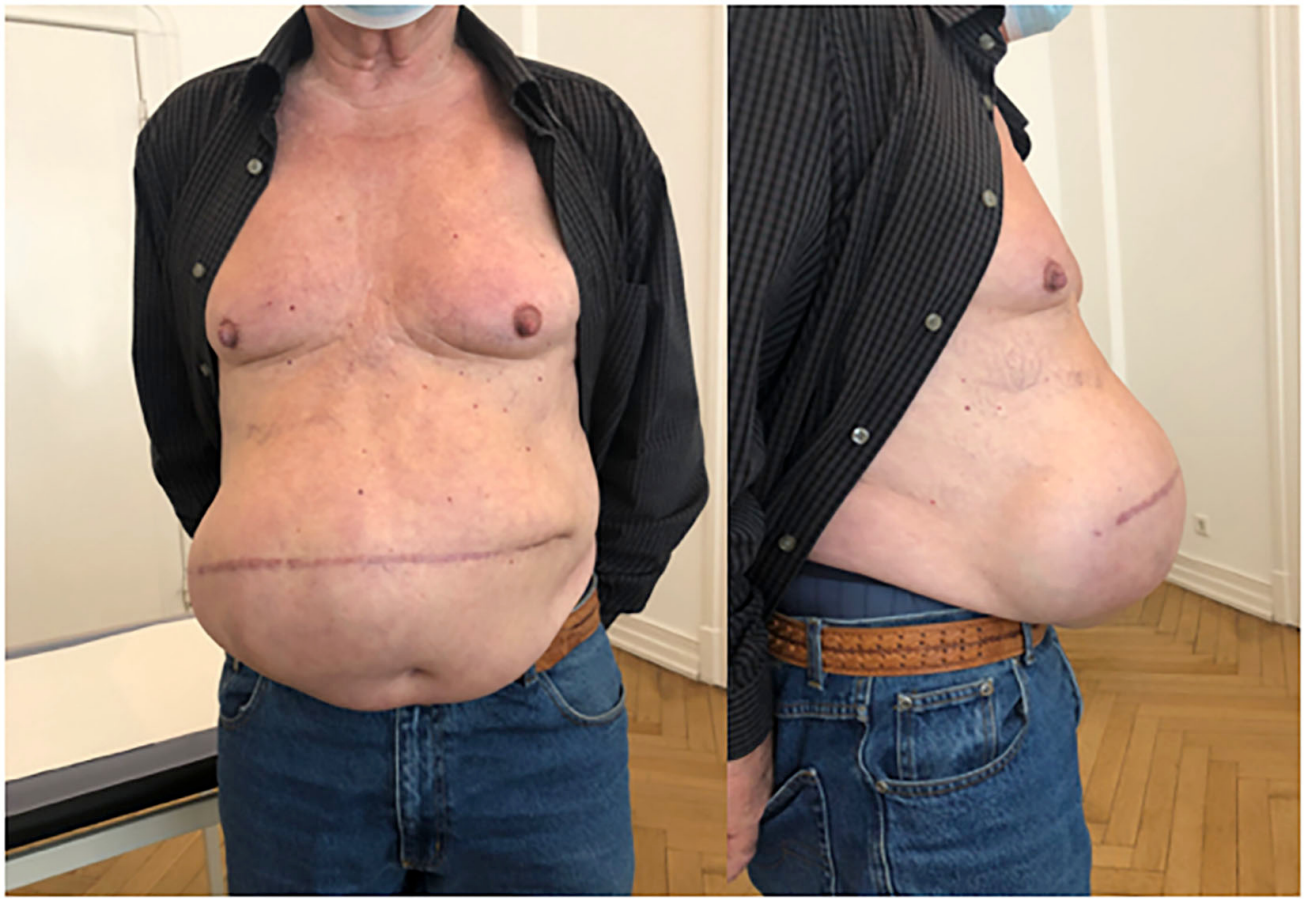
Patient 2 preoperative (transverse scar hernia), private archive Prof. Dr. H. Niebuhr 9.

**Figure 9:**
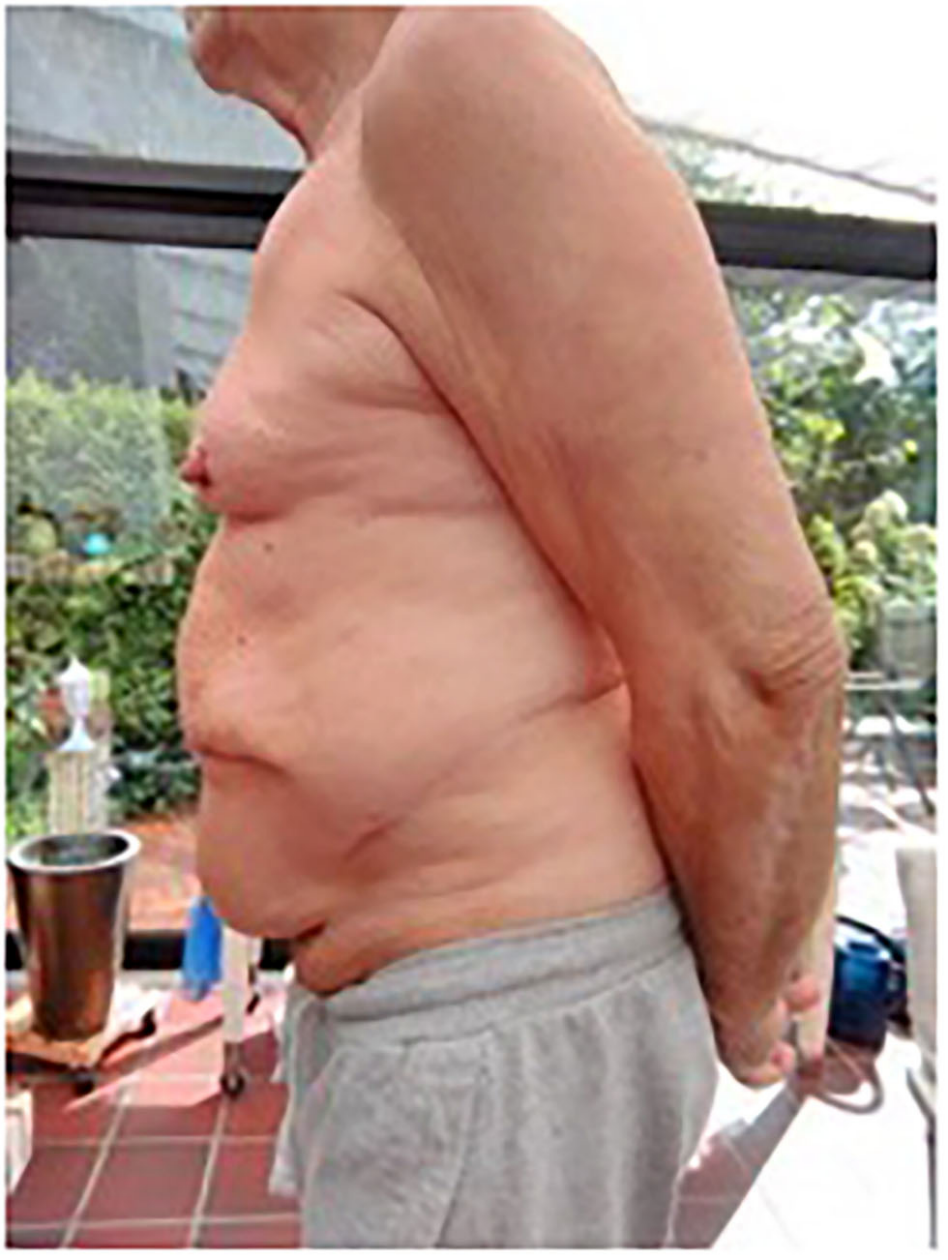
Patient 2 postoperative: lateral view of patient, private archive: Prof. Dr. H. Niebuhr 10.

The Fasciotens® Abdomen, invented by Beyer and Lill and used in this study, was developed explicitly for abdominal wall stretching in the open abdomen and large loss of domain incisional hernias (See Figure 8 before and Figure 9 after the procedure). Thus, from a legal perspective, the surgeon may use the medical device as intended, as opposed to off-label use of other techniques such as BTA application (16).

For the current indication, a technical solution where the Fasciotens® frame can be fixed externally should be a goal to avoid pressure on the patient from the device. In the current study, we combined the anteriorly-directed traction used by Eucker et al. in the AWEX method with a horizontal component to create diagonal traction. This resembles the natural forces of the abdominal wall more than purely anteriorly-directed traction. In this context, it is also important to be able to quantify the applied traction force. Although, to date, the traction method has been carried out with a subjectively adjustable force, the device used here can quantify the pull on the fascia up to 10 kg. Another improvement would be further quantification of the total traction force to significantly more than 10 kg. Standardizing the applied traction could prevent tissue trauma from excessive traction forces.

Overall, the complication rate was low (19% complications requiring revision) in a group of multimorbid patients. Immediate postoperative pain was low (average 2.5). Long-term follow-up examinations are still pending. Haskins et al. observed significant reductions in pain 6 months postoperatively compared to preoperatively in another surgical procedure (transabdominal release), which is why we also expect improvement (17). Given the complexity of these operations, the length of hospital stay (14.5 days) can also be considered low. When we exclude the outlying maximum stay of 75 days, the average length of hospital stay was 10.9 days.

## Conclusion

The intraoperative anterior-diagonal traction method represents a promising, less invasive alternative to various forms of CS, with a significant gain in fascial length (*p* < 0.001). This method enables primary closure of complex (LOD) hernias and is therefore probably less prone to complications than standard care.

A prospective multi-center study should be carried out to further evaluate the above findings.

## Limitations

Because this study was designed as an observational study, there are limitations. The lack of restrictive inclusion and exclusion criteria led to a very diverse study population. In addition, the participation of several centers should be mentioned because there were differences in pre- and postoperative care (imaging, additional chemical component separation, etc.) as well as surgical techniques, which challenges the comparability of the results. Finally, the limited follow-up period of only 6 weeks represents a substantial limitation. For the time being, for example, no statement can be made about the long-term complication rate.

## Data Availability Statement

The raw data supporting the conclusions of this article will be made available by the authors, without undue reservation.

## Ethics Statement

Ethical review and approval was not required for the study on human participants in accordance with the local legislation and institutional requirements. The patients/participants provided their written informed consent to participate in this study.

## Author Contributions

HN and TA wrote the manuscript with support from JE and input from all authors. HN, HD, WR, CP, HS, MR, MA, and FK performed the procedures. HN and JE contributed to the interpretation of the results. HN and MR designed the figures. JE devised the project and designed the study, performed the statistical analysis, and supervised the project scientifically. All authors took part in the discussion of the results leading to the final manuscript.

## Conflict of Interest

The authors declare that the research was conducted in the absence of any commercial or financial relationships that could be construed as a potential conflict of interest.
